# Engineering 2D Materials for Photocatalytic Water-Splitting from a Theoretical Perspective

**DOI:** 10.3390/ma15062221

**Published:** 2022-03-17

**Authors:** Mukesh Jakhar, Ashok Kumar, Pradeep K. Ahluwalia, Kumar Tankeshwar, Ravindra Pandey

**Affiliations:** 1 Department of Physics, Central University of Punjab, Bathinda 151401, India; mjakhar7665@gmail.com; 2 Department of Physics, Himachal Pradesh University, Shimla 171005, India; pk_ahluwalia7@yahoo.com; 3 Department of Physics and Astrophysics, Central University of Haryana, Mahendragarh 123031, India; drtankeshwar@gmail.com; 4 Department of Physics, Michigan Technological University, Houghton, MI 49931, USA; pandey@mtu.edu

**Keywords:** first-principles theory, electronic structure, 2D materials, photocatalysts, water splitting

## Abstract

Splitting of water with the help of photocatalysts has gained a strong interest in the scientific community for producing clean energy, thus requiring novel semiconductor materials to achieve high-yield hydrogen production. The emergence of 2D nanoscale materials with remarkable electronic and optical properties has received much attention in this field. Owing to the recent developments in high-end computation and advanced electronic structure theories, first principles studies offer powerful tools to screen photocatalytic systems reliably and efficiently. This review is organized to highlight the essential properties of 2D photocatalysts and the recent advances in the theoretical engineering of 2D materials for the improvement in photocatalytic overall water-splitting. The advancement in the strategies including (i) single-atom catalysts, (ii) defect engineering, (iii) strain engineering, (iv) Janus structures, (v) type-II heterostructures (vi) Z-scheme heterostructures (vii) multilayer configurations (viii) edge-modification in nanoribbons and (ix) the effect of pH in overall water-splitting are summarized to improve the existing problems for a photocatalytic catalytic reaction such as overcoming large overpotential to trigger the water-splitting reactions without using cocatalysts. This review could serve as a bridge between theoretical and experimental research on next-generation 2D photocatalysts.

## 1. Introduction

Developing clean and renewable energy resources is critical in today’s world to meet the enormous difficulties posed by global energy and environmental crises. With the long-term utilization of fossil fuels, the exhaustion of energy sources will severely affect sustainable development. Meanwhile, massive CO_2_ emissions have dramatically raised CO_2_ concentrations in the atmosphere, causing severe environmental issues such as global warming and rising sea levels. As an alternative, solar energy is becoming the ultimate energy source for all life on our planet [1,2,3,4]. 

Although the total solar energy incident on the earth in one hour is greater than the annual global energy consumption, the most pressing difficulty remains the gathering and storing of the diffused form of energy to ensure a feasible and continuous fuel supply [5]. Among the different technologies, photocatalytic process or artificial photosynthesis is a more appealing method of harnessing solar energy has piqued interest due to three key benefits (i) ability to generate O_2_ and H_2_ at different electrodes, removing the separation issue; (ii) the ability to operate in ambient circumstances; and (iii) the ability to build a system using only stable and abundant inorganic materials [6]. It is well known that solar energy is being used to run thermodynamic uphill reactions to split the water into hydrogen and oxygen [7,8]. It appears to be a straightforward method that combines water, semiconductor material, and solar light. However, each of these elements has a role in the reaction’s overall efficiency. Several factors, such as thermodynamics and kinetics, prevent this approach from realizing its full potential [9].

Fundamental knowledge of the mechanism for water-splitting photocatalysis is of great interest in developing suitable photocatalysts for large-scale industrial uses [10,11,12,13,14]. Under direct solar light, nanoscale semiconductor photocatalysts are employed to achieve reasonable reaction rates, generate charge carriers, and guarantee the surface sites for hole-mediated oxidation or electron-mediated reduction [15,16,17]. As a result, considerable effort has been expended to search for effective photocatalysts capable of producing hydrogen energy [18,19,20,21,22,23]. However, due to low solar light consumption efficiency and quantum efficiency produced by charge recombination on the catalysts surfaces, only a few show good photocatalytic activity for water splitting [24,25].

In recent years, the focus has been shifted to low-dimensional semiconductor photocatalysts to improve the rates of photocatalytic reactions [26,27]. After the discovery of graphene, there have been significant advances in synthesis and characterization of novel 2D materials, including Boron based (h-BN, borophenes) [28], Group IV (Silicene, Germanene, Stanene) [29], Group V (Phosphorene, Arsenene, Antimonene) [30], Group IVA-VIA (SnO, SnS_2_, SnSe_2_) [31], Group III-IV (GaS, GaSe) and Transitional Metal-based (Metal Carbides, Mxenes) and 2D perovskites materials [32]. Subsequently, the research into these 2D materials and their composites and heterostructures for photocatalytic water-splitting has been initiated by researchers [33,34,35].

These 2D materials exhibit the following properties: (i) high surface/volume ratio, (ii) short transport path for photo exciting holes and electrons on the surface of photocatalyst, (iii) higher conductivity which benefits charge transfer to adsorbates (iv) the improved mechanical properties which help in durability and (v) accessibility to handle in production as well as the recycling process [17,36]. Most of these 2D materials are semiconductors with a bandgap between 1–3 eV, meeting the essential requirements of an energy band gap and adequate band alignment for efficient absorption of a wide portion of the solar spectrum with photogenerated redox potentials [37,38]. Thus, the fundamental physical and chemical features of these 2D semiconducting materials have led to their significant role in photocatalytic applications [39,40,41]. However, the experimental synthesis of 2D materials requires highly skilled researchers and expensive equipment. Besides, experimentalists do not exactly know which 2D monolayers are suitable candidates until they can fabricate them.

On the other hand, computational techniques have recently emerged as an alternative strategy for screening catalysts for specific reactions, with high selectivity, efficiency, and low cost, which directs the selection of elements used to steer the photocatalyst discovery [42]. This method has the advantage of allowing us to study the sensitivity of the fundamental parameters and identify the essential qualities in determining overall photocatalytic efficiency. These theoretical methods can offer a reasonable estimation of quantum and solar-to-hydrogen efficiency as a function of wavelength. Furthermore, absorption coefficients, band positions, and other properties such as carrier concentrations, effective masses, dielectric constant, mobility, and lifetime have been predicted, i.e., we are one step closer to “photocatalysis by design” owing to modelling [12,14].

The density functional theory (DFT) computation is widely used to estimate the properties of materials and the various components in the overall reaction cycle for photocatalysis [14,43,44,45]. The standard local density approximation (LDA) and generalized gradient approximation (GGA) functionals are known to underestimate bandgaps [46]. However, the Heyd–Scuseria–Ernzerhof (HSE06) hybrid functional predicts more precise findings matching the experiments [47,48,49,50]. Most of the theoretical predication of electronic structures for water splitting is calculated using the HSE06 hybrid functional. Furthermore, the light adsorption computed using the HSE06 approach can only be used as a reference while looking for photocatalysts under the one-particle assumption. While the excitonic effects are considered through the GW and Bethe-Salpeter equation (BSE) methods which yields an accurate light absorption spectrum [51,52,53,54].

DFT calculations have made significant contributions to a deep understanding of electronic behavior and structure-performance relationships for photocatalytic materials. Recently, several great review studies on DFT calculations of semiconductor-based photocatalysts have been published [12,14,41,55]. Theoretical efforts have primarily focused on the first step in bandgap engineering, which includes doping foreign elements and developing solid solutions because photoexcited electrons and holes are generated only when the photocatalyst’s bandgap is lower than the incident photon energy. Moreover, Henderson et al. summarized the theoretical developments of the relationships between a surface’s properties and various observed photo-initiated events occurring at TiO_2_ during photocatalysis and explored the molecular-level understanding of the electron transfer dynamics and mechanistic aspects [56]. Furthermore, in 2013, Liao and Carter suggested strategies to improve the photocatalytic process, such as semiconductor light absorption, electron and hole migration, material band-edge alignment, and surface reactions. They also show the limitation of present simulation and modelling approaches on computing surface reactions, photogenerated carriers, and kinetic barriers [57]. Akimov et al. summarized the theoretical methodologies used to examine the dynamics of photogenerated charge separation, transport, relaxation, and recombination at metal oxide surfaces [58].

Cai and Feng presented the theoretical contributions to understanding charge transfer in TiO_2_ composite systems. They analyzed how charge transfer affected band bending, electron-hole separation, mass transport at the surface or interface, and surface catalysis [59]. Several topical reviews describing first-principles methodologies relevant to the study of solid-liquid interactions have been reported [14,39,60]. In addition, some groups recently evaluated theoretical achievements in two-dimensional materials for photocatalytic water-splitting [11,40,61,62,63].

In this review, water-splitting studies have been used as specific examples to understand the fundamental theory of reactions occurring on a photocatalyst’s surface. The mechanisms of overall water-splitting have been explained briefly. The characteristics and processes that affect the kinetics and thermodynamics of surface reactions are also discussed, focusing on the role of overpotential in the water-splitting mechanism. Then the approaches for improving photocatalytic activity by design: (i) single-atom catalysts (SACs), (ii) defect engineering, (iii) strain engineering, (iv) Janus structures, (v) type-II heterostructures, (vi) Z-scheme heterostructures, (vii) multilayer configurations, (viii) edge-modification in nanoribbons, and (ix) the effect of pH in overall water-splitting to overcome large overpotential, have been discussed in detail. Finally, conclusions, future perspectives, and challenges are provided.

### 1.1. Overall Water Splitting

The water-splitting reaction is endothermic in which water is broken down into oxygen and hydrogen:

(1) $$2H_{2}O\to 2H_{2}+O_{2}$$

Splitting water into hydrogen is an uphill chemical process with a corresponding rise in Gibbs free energy (ΔG° = 237 kJ mol^−1^) [64].

In general, the overall water-splitting photocatalysis at the semiconductor catalyst interface has three significant steps: (i) photons with adequate energy absorption (which is larger than the bandgap) on the surface of a photocatalyst, which can accelerate excited electrons and holes for subsequent redox reactions, (ii) the separation and migration of charges on the photocatalyst’s surface in a brief period, and (iii) surface reactions, such as HER (i.e., hydrogen evolution reaction) and OER (i.e., oxygen evolution reaction) for water reduction (Equation (2)) and oxidation (Equation (3)) on the surface of photocatalysts, respectively.

(2) $$2H^{+}+2e^{-}\to H_{2}$$

(3) $$H_{2}O+2H^{+}\to 2H^{+}+1/2O_{2}$$

Significant potential losses are expected (“interfacial loss”), resulting from entropy contributions of electrons and potential interfacial barriers caused by poor alignment [65,66]. The efficient electron/hole transport to the photocatalyst adjusts the potentials either negatively or positively at short time scales of milliseconds to seconds. It then maintains steady-state potentials that allow steady-state electrochemical redox processes to produce H_2_ and O_2_ [12].

However, the photocatalytic reactions follow a complex competing sequence for multistep processes. Lighting the preceding physical features determine which factor(s) dominates the net photocatalytic activity. This emphasizes the significance of comprehending the kinetics and dynamics of a catalytic reaction to build realistic methodologies for the rapid development of photocatalysis in the future.

#### Thermodynamic Requirements

**Figure 1:**
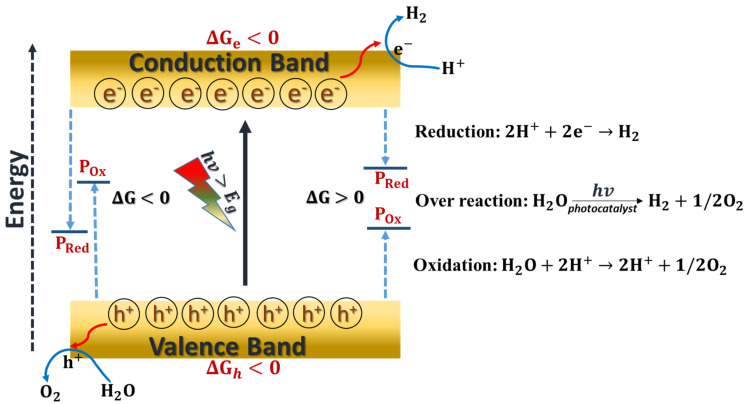
A schematic diagram of the band alignment of a semiconductor photocatalyst.

The photocatalytic materials, typically consisting of metal oxide semiconductors, efficiently absorb visible light irradiation up to a wavelength of 520 nm to achieve around 10% solar to hydrogen (STH) efficiency [67,68]. Such semiconductors must meet the thermodynamic conditions for driving water breakdown into H_2_ and O_2_ [9]. The photocatalysis mechanism can generally be characterized by the electronic band structure, as shown in Figure 1. A photon with an energy higher than the bandgap excites an electron from a filled valence band state to an empty conduction band. After photo-excitation, the ability of a photoexcited electron (e^−^) and hole (h^+^) to reduce and oxidize is then determined by the bottom of CB and top of VB in the band structure. Those charge carriers should be thermally relaxed to the bottom of the CB and the top of the VB. At the bottom of CB, the conditions for a photocatalytic reaction should be higher (more positive) than the reactant’s reduction potential (P_red_).

The top of VB is suggested to be more positive than the oxidation potential of the reactant to be oxidized (P_ox_). Since the difference between P_red_ and P_ox_ corresponds to the total Gibbs energy change (ΔG), the ΔG is negative when P_red_ is lower than P_ox_ and vice-versa. Importantly, to drive a chemical reaction, ΔG must be negative in the reduction and oxidation steps by e^−^ and h+, which is the determining factor to initiate a photo redox process. Thus, the photocatalytic processes are regulated by partial ΔGs, i.e., $\Delta G_{e}$ and $\Delta G_{h}$ shown in Figure 1.

Moreover, photocatalysis may be used to generate both positive and negative ΔG reactions [69,70]. For example, ΔG < 0 is found in the photo-degradation of organic molecules in the presence of oxygen. On the other hand, ΔG > 0 occurs for water splitting [13,15] and CO_2_ reduction [71] with substantial positive changes in ΔG. The material does not affect the thermodynamics of the process but only modifies the kinetics by generating new reaction pathways through absorption of optical light [72].

### 1.2. Reaction Kinetics

The photocatalytic reaction occurs when a photocatalyst satisfies the thermodynamic requirements of a particular redox process. It is possible that the reaction will occur and the chemical reaction rate can be forecast by their kinetics. Various kinetic models have been developed [73]. The production and migration of electron-hole pairs within the surface and recombination of electron-hole pairs followed by the oxidation and reduction reaction with reactants take place on the surface of photocatalyst materials. In general, light irradiation generates electron-hole pairs, where surface defects can trap holes or oxidize the reactant. In contrast, electrons move arbitrarily via a multi-strapping pathway until they come across a recombination centre where they can recombine with holes or an active site, and the surface chemical reaction can be completed [69,70,74].

The elementary steps of calculating the Gibbs free energies (ΔG) for catalytic reactions using the standard hydrogen electrode (SHE) model are given by Norskov et al. [75,76].

(4) $$\Delta G=\Delta E+\Delta E_{\mathrm{ZPE}}-T\Delta S+\Delta G_{U}+\Delta G_{\mathrm{pH}}$$

where $\Delta E_{\mathrm{ZPE}}$ and TΔS denotes the zero-point energy difference and entropy, respectively, and ΔE is the intermediates absorption energy. The relevant electrode potential U is $\Delta G_{U}$ = −eU. The ΔG was corrected to H^+^ concentrations and represented by $\Delta G_{\mathrm{pH}}$.

Two electron paths are involved in the HER process, including a fast proton/electron transfer step and a fast hydrogen release step:

(5) $$*+H^{+}+e^{-}\to H^{*}$$

(6) $$H^{*}+H^{+}+e^{-}\to *+H_{2}\left(g\right)$$

where * and H* represent the catalyst’s active site and adsorbed H^+^ ions, respectively.

It is well-known that photocatalytic water oxidation for oxygen evolution is complex and challenging. The Norskov group has done substantial theoretical research on the OER mechanism [76,77,78,79,80]. The electrochemical OER’s proposed mechanism included four proton-coupled electron transfer (PCET) steps:

(7) $$H_{2}O+*\;\to \mathrm{HO}*+H^{+}+e^{-}$$

(8) $$\mathrm{HO}*\;\to O*+H^{+}+e^{-}$$

(9) $$H_{2}O+O*\;\to \mathrm{HOO}*+H^{+}+e^{-}$$

(10) $$\mathrm{HOO}*\;\to O_{2}+*+H^{+}+e^{-}$$

where * stands for the coordinatively unsaturated sites where the reaction takes place and OH*, O*, and OOH* denote the adsorbed intermediates. Each step must be supplied with enough energy to drive the process, which is fulfilled by the external electrode energy according to Norskov’s calculations. The energy term qU was used to empirically reflect this process, which derives from the electrons’ higher energy, arising from the applied voltage.

The OER processes for a given applied voltage bias can be produced energetically and thermodynamically favourable (ΔG < 0). The reaction may contain kinetic barriers associated with each step and be excluded from the reaction-free energy calculations. According to first-principles estimates, the rate-determining step can vary based on chemical circumstances, calculation methods, and substrate composition [81]. Theoretical calculations and spectroscopic measurements for detecting intermediates during catalytic reactions are informative and helpful in determining the mechanism of photocatalytic water-splitting.

## 2. Quantification of Essential Photocatalytic Water Splitting Properties

Apart from the significance of the kinetics and dynamics of a photocatalytic reaction, many other vital parameters involved in the process of photocatalytic reactions are (i) potential of the reaction, (ii) exciton separation, and (iii) Solar-to-Hydrogen (STH) efficiency, which depicts the photocatalytic water-splitting process sequentially occurring at different time scales.

### 2.1. Overpotential

**Figure 2:**
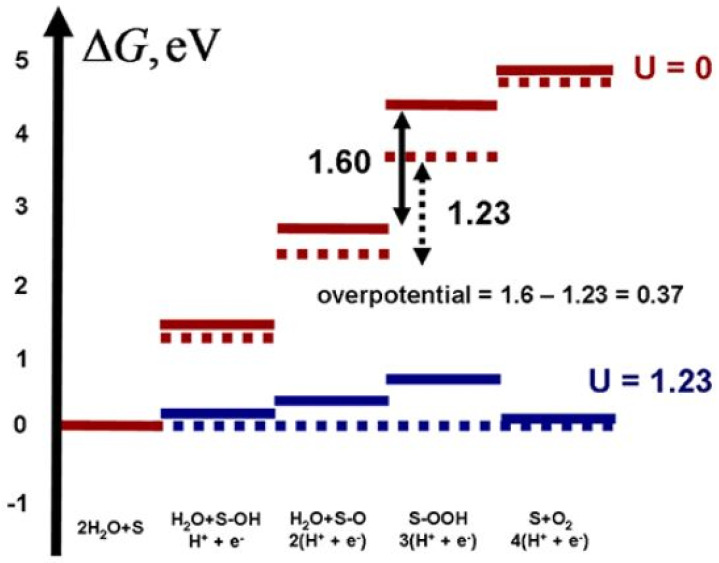
The free energy profile for the OER on TiO_2_. Reprinted with permission from ref. [58]. Copyright 2013 ACS.

The HER and the OER require an external energy source such as solar radiation, which generates an electric current involving electrons and holes. The electrode potential (U) represents the external energy in an electrochemical cell, which reduces the free energy barrier and causes thermodynamic and kinetic transformations. Norskov’s group investigated the OER’s thermodynamics by estimating the free energy change (ΔG) of the products and reactants in each step using one-by-one electron transfer steps as shown in a typical free energy diagram (Figure 2).

Water oxidation $E_{O_{2}/H_{2}O}^{0}$ has a standard electrochemical potential of 1.23 eV versus NHE. This value corresponds to the red dashed lines in Figure 2, representing the ideal free energy profile for a 4-e oxidation process. Each of the four consecutive steps in this idealistic scenario continues with a constant ΔG equal to the usual electrochemical potential. As defined by Equations (7)–(10), the various steps of the OER have distinct free energy change values in the entire system (solid red lines in Figure 2). These aberrations can be associated with the conformational and electronic changes of the reacting subsystem and how such reactions impact the surroundings, such as the solvent and substrate. The third step given by Equation (9) is the rate-limiting step with 1.60 eV potential. As a result, the electrode potential (1.23 eV) is inadequate to allow the thermodynamic oxidation process to occur (solid blue line). At U = 1.23 V, the energy diagram flattens out (Figure 2, dotted blue line) and permits all steps to be thermodynamically feasible. The minimum voltage supplied to the electrode requires at least 1.60 V, which is the highest ΔG in the reaction pathway (rate-limiting potential). The 0.37 V external voltage is required for the optimal procedure (1.60 V − 1.23 V = 0.37 V). This extra voltage is called the overpotential (η). The theoretically estimated overpotential for TiO_2_ ranges from 0.76 to 0.78 V [76], close to the experimental results of 0.9–1.1 V [82].

#### Band Alignments and Overpotentials of 2D Materials

**Figure 3:**
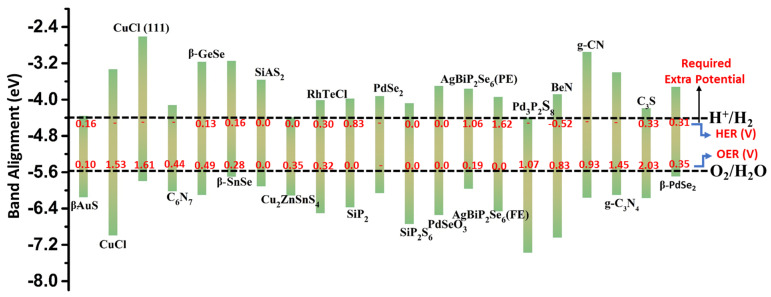
Band alignments and required extra potential of some typical 2D materials concerning the water reduction and oxidation potential for photocatalytic water-splitting.

The calculated band edge position of VBM (valence band maxima) and CBM (conduction band minima) for some typical 2D materials concerning the water reduction and oxidation potentials for photocatalytic water-splitting is shown in Figure 3. The overpotential and required extra potential to trigger photocatalytic water-splitting OER and HER reactions employing 2D materials are listed in Table 1 and Table 2, respectively.

Note that the presence of appropriate band edge positions does not always assure that the material will be an effective photocatalyst for overall water-splitting. We do not know whether photo-generated electrons and holes in the monolayer provides sufficient driving force to activate overall water-splitting. Due to the energy loss during the photogenerated carriers’ migration between different materials [83], the overpotential is a crucial element for evaluating the performance of photocatalysts for water splitting [84]. To this end, further research into the processes of both the HER and OER is required to better understand the monolayer’s photocatalytic activity. For example, monolayers of C_3_S, CuCl (111), CuCl, g-C_3_N_4_, and Pd_3_P_2_S_8_ exhibit a very high overpotential (rate-limiting potential) in the oxygen evolution half-reaction (Table 1). 

The potential provided by the photogenerated holes (U_h_: the difference in energy between the VBM and the hydrogen reduction potential) cannot overcome the high overpotentials, resulting in high required external potential (>1 V) to proceed with the oxygen evolution reaction for water splitting (Table 1 and Table 2). While g-CN, BeN, β-GeSe, C_6_N_7_, and Cu_2_ZnSnS_4_ (112) required the external potential greater than 0.40 V. The β-PdSe_2_, AgBiP_2_Se_6_ (PE), RhTeCl, Cu_2_ZnSnS_4_, β-SnSe, and β-AuS monolayers required minimal external potential to process the OER reaction. Remarkably, under the external potential provided by photogenerated holes, the AgBiP_2_Se_6_ (FE), SiP_2_S_6_, PdSeO_3_, SiP_2_, and SiAS_2_ monolayers can proceed OER without using sacrificial reagents or cocatalysts.

**Table 1:** Overpotential and required external potential for HER and OER for 2D materials.

2D Materials	HER Over Potential	HER Required External Potential	OER Over Potential	OER Required External Potential	Ref.
Cu_2_ZnSnS_4_ (112)	0.74	-	1.73	0.50	[85]
Cu_2_ZnSnS_4_	0.0	-	1.58	0.35	[85]
GaAs	0.0	0.0	2.65	1.39	[86]
C_3_N_5_ bilayer	0.74	0	1.94	0.69	[87]
Penta-SiAs_2_	0.72	0	1.45	0	[88]
β-GeSe	1.39	0.13	2.56	0.49	[89]
BeN_2_	0.72	−0.52	2.06	0.83	[90,91,92]
β-SnSe	1.44	0.16	1.95	0.28	[89]
β-AuS	0.24	0.16	2.22	0.10	[93]
PdSeO_3_	0.98	0	1.63	0	[94]
AgBiP_2_Se_6_ (PE)	1.33	1.06	2.12	0.19	[95]
AgBiP_2_Se_6_ (FE)	1.71	1.62	2.17	0	[95]
PdSe_2_	1.17	-	2.15	-	[96]
LiGaS_2_ bilayer	1.15	0	2.08	0	[97]
RhTeCl	1.14	0.30	1.55	0.32	[98]
CuCl	0.95	-	2.76	1.53	[99]
CuCl (111)	−0.56	-	2.84	1.61	[99]
C_6_N_7_	0.56	-	1.67	0.44	[100]
Pd_3_P_2_S_8_	-	-	2.77	1.07	[101]
SiP_2_S_6_	0.12	0	2.000	0	[102]
g-CN	1.15	-	2.16	0.93	[103]
g-C_3_N_4_	-	-	2.68	1.45	[104]
β-PdSe_2_	1.54	0.31	1.58	0.35	[105]
C_3_S	0.33	0.07	3.76	2.03	[106]
SiP_2_	1.7	0.83	1.50	0	[107]
Janus WSSe	0	0.58	2.39	0	[108]
Janus Pd_4_S_3_Se_3_	0.77	-	2.99	1.76	[109]
Janus Pd_4_S_3_Te_3_	0.18	-	2.50	1.27	[109]
Janus Pd_4_Se_3_Te_3_	0.73	-	2.83	1.60	[109]

**Table 2:** Overpotential and required external potential for HER and OER for 2D heterostructures.

2D Heterostructure	HER Over Potential	HER Required External Potential	OER Over Potential	OER Required External Potential	Ref.
PtS_2_/Are	0	0	2.00	0	[110]
P_4_O_2_/Black Phosphorus	0.85 β-site	0.0	2.65 β-site	0.0	[111]
P_4_O_2_/Black Phosphorus	1.11 α-site	0.04	3.15 α-site	0.33	[111]
In_2_SeS/g-C_3_N_4_	-	-	1.56	0.74	[112]
AlP_3_−GaP_3_	-	-	1.65 P1 site	0.37	[113]
CuInP_2_S_6_/Mn_2_P_2_S_6_	1.68	0.14	2.36	0.13	[114]
arsenene/g-C_3_N_4_	-	-	2.72	1.03	[115]
C_2_N/GaTe	-	-	2.70	1.47	[116]
C_2_N/InTe	-	-	2.17	0.94	[116]
MoSe_2_/SnSe_2_	−0.24	0	2.06	0	[117]
WSe_2_/SnSe_2_	−0.23	0	2.04	0	[117]
C_2_N/WS_2_	-	-	3.04	1.81	[118]
GeSe/SSn	0.8	0	1.94	0	[119]

For HER, the monolayer AgBiP_2_Se_6_ (FE) and AgBiP_2_Se_6_ (PE), overpotential cannot be overcome by the potential provided by the photogenerated electrons (U_e_: the difference in energy between the hydrogen reduction potential and the CBM) as shown in Table 1. Therefore, these materials required a potential >1 to trigger the reduction reaction. The monolayer SiP_2_ and AgBiP_2_Se_6_ (FE) efficiently activate only the oxidation reaction. In contrast, the Cu_2_ZnSnS_4_, C_3_N_5_ (2L) GaAs monolayer can trigger only the reduction reaction without the help of additional cocatalysts. Notably, very few monolayers (Penta-SiAs_2_, PdSeO_3_, SiP_2_S_6_, LiGaS_2_ (2L)) can be used as a high-performance photocatalyst for overall water-splitting, triggering both oxidation and reduction reaction without using any cocatalysts due to the sufficient external potential provided by photogenerated carriers.

### 2.2. Exciton Binding Energy

The electron-hole pairs will be formed as the photon is absorbed, and they must be separated appropriately to ensure adequate free photoexcited carriers for the subsequent water redox reactions. The exciton binding energy plays a vital role in examining the carrier separation induced by photo-excitation, and it is defined as: $E_{b}=E_{\mathrm{QP}}-E_{\mathrm{OPT}}$, where $E_{\mathrm{OPT}}$ and $E_{\mathrm{QP}}$ stand for the energy at the first optical absorption peak and quasiparticle bandgap, respectively. The smaller the value of $E_{b}$, the easier it is to separate the carrier. The minimum energy needed to ionize an exciton from its lowest energy state is given by $E_{b}$ [12,120]. The effective masses and dielectric constant are two crucial parameters that influence the values of the exciton binding energy.

Currently, DFT calculations and beyond can accurately predict exciton binding energies, effective masses, and different crystal orientations. The significant distortion causes an anisotropic electronic field during exciton formation, facilitating charge separation [121]. Furthermore, these quasiparticles are sensitive to a range of external stimuli, which may be used to alter 2D materials’ intrinsic optical and optoelectronic properties, making them intriguing candidates for novel optoelectronic applications.

**Figure 4:**
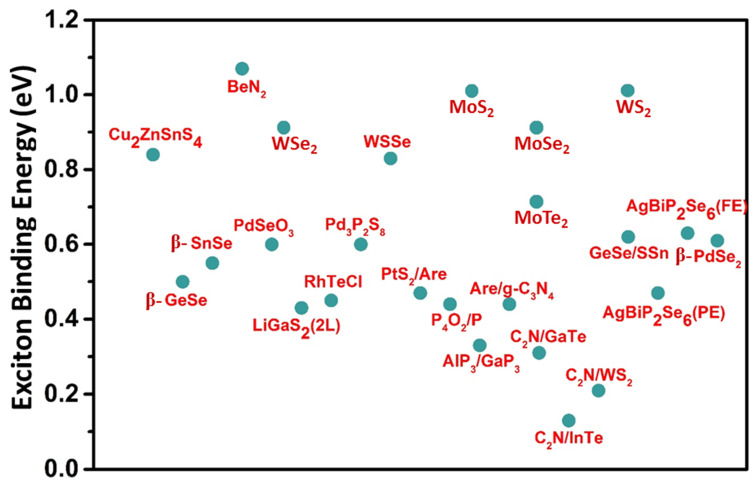
The calculated exciton binding energy of 2D materials.

A representative scheme of exciton binding energy for various 2D responsive materials is shown in Figure 4. In the chalcogen-based 2D materials, the exciton binding weakens as the chalcogen becomes heavier, explaining the enhanced dielectric screening provided by heavier chalcogens’ to diffuse more orbitals. The amplitude of the spin-orbit splitting is directly related to the exciton splitting in these materials, and the exciton splitting in WX_2_ compounds is substantially higher than in MoX_2_ compounds. Excitation energies for Molybdenum and Tungsten dichalcogenides materials are predicted to range from 1 to 2 eV, suggesting potential applications in the near-IR to the red regime [122]. As seen in Figure 4, the $E_{b}$ of Janus, WSSe is less than monolayer WS_2_ and WSe_2_ due to the built-in electric field directed from the Se layer to the S layer [108,122]. Moreover, the excitation energies of C_2_N/WS_2_ heterobilayer are less than their monolayer counterparts [118].

### 2.3. Solar-to-Hydrogen (STH) Efficiency

STH efficiency is an important characteristic that can be used as a realistic standard for measuring the performance of photocatalysts. The STH efficiency has been estimated to evaluate the photocatalytic performance of the monolayer, assuming 100% efficiency of the catalytic reaction [123,124]. The STH efficiency (Q_STH_) for the semiconductor can be determined by [84,111]:

(11) $$Q_{\mathrm{STH}}=\frac{\Delta G\int_{E_{g}}^{\infty }\frac{P\left(\hbar \omega \right)}{\hbar \omega }d\left(\hbar \omega \right)}{\int_{0}^{\infty }P\left(\hbar \omega \right)d\left(\hbar \omega \right)+\Delta \phi \int_{E_{g}}^{\infty }\frac{P\left(\hbar \omega \right)}{\hbar \omega }d\left(\hbar \omega \right)}$$

where ΔG = 1.23 eV and P(hω) represents the potential difference for water splitting and the AM1.5 solar energy flux at the photon energy of hω, respectively.

The ultra-violet (UV) photocatalysts perform better than visible light photocatalysts for hydrogen generation via solar water splitting because UV light has higher photonic energy than visible light. Most of the described photocatalysts are only active when exposed to UV light. However, ultraviolet light (400 nm) accounts for only 4%, while visible light (400–800 nm) and infrared light (>800 nm) contribute about 53% and 43%, respectively, of total solar energy. Since UV light accounts for only a small amount of solar energy, it is vital to rationally design and build photocatalysts that can harvest more visible or infrared light, effectively boosting the low STH conversion efficiency throughout a broad spectral range. Thus, a less efficient photocatalyst that absorbs visible light is preferred than a more efficient photocatalyst that absorbs only UV light [12,125,126].

**Figure 5:**
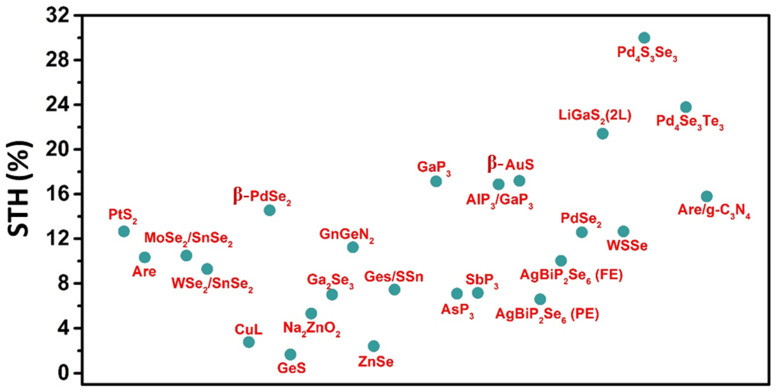
Solar-to-hydrogen (STH) efficiency for 2D materials.

Moreover, heterostructures are created by stacking light absorbers with various bandgaps on top of one another, enhancing photo potential and exploiting a more significant portion of the solar spectrum. These systems can create more photo potential with a broader range of solar absorption, resulting in more excellent STH conversion efficiencies [125,126]. The light absorption efficiency is strongly dependent on the bandgap. A representative scheme of STH for various 2D responsive materials is shown in Figure 5, where the STH range for mostly 2D materials is from 4–20%. In contrast, Janus Pd_4_S_3_Se_3_ shows the highest STH of 30.01%.

## 3. Engineering of 2D Materials for Enhanced Photocatalytic Surface Activity

### 3.1. Single-Atom Catalysts (SACs)

**Figure 6:**
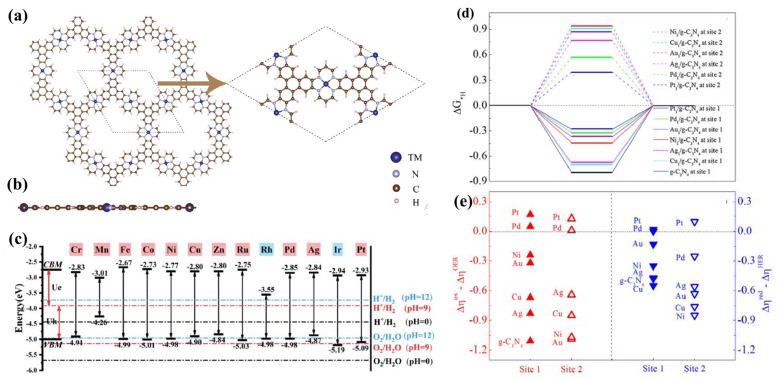
(**a**) Top and (**b**) side views of the planar TM-TAA MOF monolayers. The rhombus primitive cell of the 2D MOFs is also shown. (**c**) Electron energies of various TM-TAA MOFs. The vertical bars show the bandgap. The vacuum level has corrected the energy level positions. Reprinted with permission from ref. [128]. Copyright 2021 RSC. (**d**) The relationships of the calculated overpotentials (Δη_OER_ and Δη_HER_) with the differences between the redox potentials of specimens and water oxidation/H+ reduction (Δη_ox_ and Δη_red_). (**e**) The free energy charges of HER at sites 1 and 2 of specimens. Reprinted with permission from ref. [104]. Copyright 2018 Elsevier.

SACs have shown tremendous promise in photocatalytic water-splitting because of distinctive geometric and electronic configurations that enhance mass and charge transfer during the photocatalytic process. The interaction of the single metal atom with the pedestal layer can affect the photocatalytic pathways and catalytic performances [127]. First-principles calculations [128] have recently demonstrated that the 2D metal-organic frameworks (MOFs) containing transition metal (TM = Cr–Zn, Ru–Ag, Ir, and Pt) atoms and tetraaza annulene (TAA) [14] can act as multi-functional photocatalysts. As illustrated in Figure 6a,b, the unit cell of two-dimensional TM-TAA MOFs has three equivalent TM atoms; each bind to four N-atoms to create nitrogen-coordinated metal macrocycles (TM-N4). The VBM and CBM of these TM-TAA MOFs estimated using the HSE06 functional were aligned relative to the vacuum level with hydrogen reduction and water oxidation (Figure 6c).

Moreover, the calculated catalytic performance of TMTAA MOFs with TM = Ru–Ag, Ir, Pt predict them to be an efficient catalyst for OER with the overpotential of 0.45 V [128]. While Rh-TAA MOF can catalyze OER with the overpotential of 0.40 V. These values are comparable or even lower than that of the best catalysts for water splitting (Pt/RuO_2_) [129,130,131]. Remarkably, under visible light irradiation, the Ir-TAA and Rh-TAA MOF monolayers could operate like very effective photocatalysts for overall water-splitting.

However, the g–C_3_N_4_ has good solar utilization and redox potentials, and it has poor carrier separation because the VBM and CBM are positioned in the closed N and C atoms [132]. We note that the recombination rate of photogenerated electron-hole pairs and overpotentials in OER and HER must be controlled to obtain high catalytic efficiency. For the above purpose, the single-atom embedded g–C_3_N_4_ has recently displayed a unique charm in oxidation [133,134] and water splitting [135] in both experiment and theory due to the high utilization rate and selectivity of SACs [136,137,138].

The absorption energy of OH, O, OOH, and H intermediates on different sites of catalysis surface is also an essential factor to determine the catalytic efficiency. TM_1_/g–C_3_N_4_ specimen shows that the adsorption energies of intermediates decrease significantly compared to pristine g–C_3_N_4_. A relationship of the overpotential with the difference between the water oxidation/H^+^ reduction and redox potential of the specimen (Figure 6d) shows that the ΔG_*H_ values are negative at site 1 (the centre of the triangular pore) and positive at site 2 (top of TM_1_ atom), suggesting a preference of adsorption of H atom on-site 1 [104]. A similar variation tendency is found for the overpotential for HER (Δη_HER_). Comparing the Δη_HER_ with the differences of reduction potentials between H^+^ reduction (Δη_red_) and specimens (see Figure 6e), the Δη_red_ of Pd_1_/and Pt_1_/g–C_3_N_4_ are equal or more significant than the Δη_HER_ of them at both absorption sites. As a result, the Pd_1_/and Pt_1_/g–C_3_N_4_ catalysts can be used to produce hydrogen [104].

In conclusion, the interaction of single metal atoms with a 2D surface impacts photocatalytic pathways and catalytic performance [127]. For example, metal atoms interacting with the g-C_3_N_4_ surface can change the material’s inherent band structure, affecting light absorption, charge carrier separation, and reaction kinetics. The development of atomically dispersed active sites is attributed to the contact between an isolated metal atom and the surface, improving the catalytic activity and stability of SACs for photocatalytic water-splitting [104,139].

### 3.2. Defects Engineering

Engineering the morphologies of photocatalysts by constructing defects, heteroatom doping, and surface/interface building is a promising strategy for improving photocatalytic performance [140,141,142,143,144,145,146]. Material with defects may be relatively more conducive to photocarrier separation, allowing more photoelectrons and holes to engage in the reduction and oxidation reactions [142]. Defects are also important in photocatalysis because they provide active sites for reaction participation, which improves light harvesting and charge carrier concentration with better charge separation [140].

**Figure 7:**
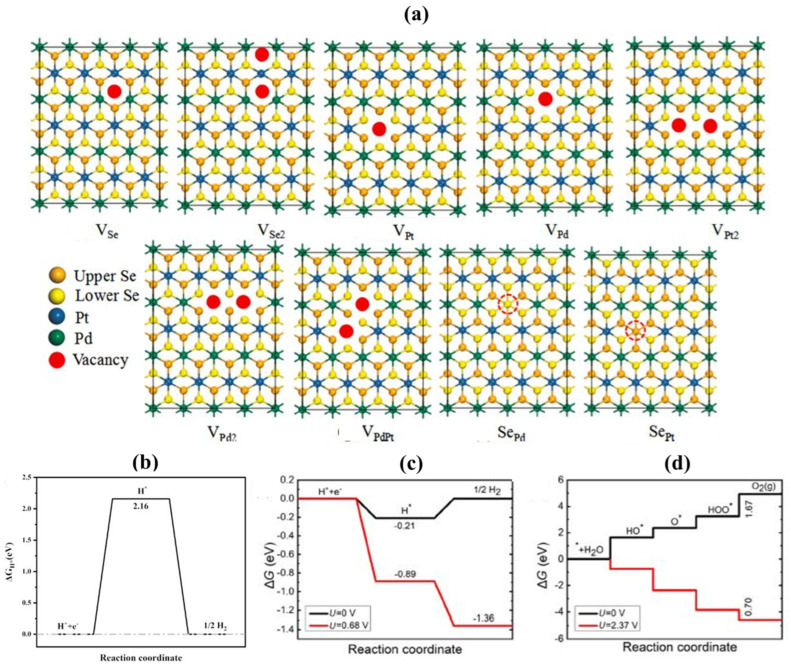
(**a**) The schematic diagrams of nine-point defects (V_Se_, V_Se2_, V_Pt_, V_Pd_, V_Pt2_, V_Pd2_, V_PdPt_, Se_Pt_, Se_Pd_) in PdPtSe_4_ monolayer. Red dots represent the defect sites. Reprinted with permission from ref. [149]. Copyright 2021 Elsevier. (**b**) Free energy profile for the 2e pathways of hydrogen reduction reactions in the Janus InGaSSe bilayer under pH = 0 and U = 0 V. The 2e and 4e for (**c**) HER and (**d**) OER pathways in the Janus InGaSSe bilayer with Ge defect under different conditions. Reprinted with permission from ref. [150]. Copyright 2018 Elsevier.

Zheng et al. [147] induced the intrinsic point defects (selenium vacancy (V_Se_), diselenium vacancy (V_Se2_), platinum vacancy (V_Pt_), diplatinum vacancy (V_Pt2_), and anti-site defects with a Se atom substituting a Pt atom (Se_Pt_)) in ultrathin PtSe_2_ layers using first-principles computations and STM/STS (Scanning Tunneling Microscopy/Scanning Tunneling Spectroscopy) [148], which can improve the photocatalytic water-splitting efficiency of PtSe_2_ monolayers [147]. Moreover, on the other side, Ma et al. [149] recently introduced Pd dopant and vacancies into PtSe_2_ to boost the basal plane for the HER. They constructed five (V_Se_, V_Pt_, V_Se2_, V_Pt2_, and Se_Pt_) and nine (VSe, V_Se2_, V_Pt_, V_Pd_, V_Pt2_, V_Pd2_, V_PdPt_, Se_Pt_, Se_Pd_.) intrinsic defect in PtSe_2_ and PdPtSe_4_ monolayer, respectively (Figure 7a). The divacancy sites (V_Se2_ and V_Pt2_) in pristine PtSe_2_ monolayers, as well as the vacancy (V_Se_, V_Pd_) and divacancy sites (V_Pt2_, V_PdPt_) in PdPtSe_4_ monolayers, significantly boost catalytic activity comparable to that of Pt [149].

More excitingly, the defect engineering significantly reduces the overpotentials of the HER and the OER to levels exclusively provided by photogenerated carriers [151,152,153,154]. For example, the overpotential of HER according to free energy calculations on the InGaSSe bilayer is about 2.16 V (Figure 7b). This calculated value is higher than photogenerated electrons potential (U_e_ = 0.68 V), implying that HER cannot be activated solely by photo-generated electrons in the InGaSSe bilayer. To improve the InGaSSe’s monolayer performance, the surface defects, including Ga vacancy, are introduced, leading to a decrement of HER overpotential to −0.21 V. Therefore, the results suggest that HER can be carried out by photogenerated electrons under photogenerated potential, as shown in Figure 7c. On the other hand, OER for the defective InGaSSe bilayer, the free energy profile becomes favourable with ΔG < 0 under the external potential provided by the photogenerated holes, indicating that the OER can proceed under these conditions (Figure 7d). As a result, vacancy defects can lower the HER and OER overpotentials of 2D semiconductors to the levels generated by photogenerated carriers, allowing overall water-splitting without reagent sacrifice.

### 3.3. Strain Engineering

By varying the distances between surface atoms, lattice using either tensile or compressive strain can fine-tune the electronic structure of surfaces [155,156]. Since the applied strain usually varies with the ions’ specific bonding and local symmetry, there are no general rules for the strain effect on the material’s electronic structure. Single-layer materials can sustain high mechanical strain [157,158]. For example, graphene can endure a 25% uniaxial strain [159], while phosphorene can sustain a 30% uniaxial strain [160]. This gives much flexibility when it comes to electronic structure engineering. This surface tuning strategy is another practical way to manipulate the catalytic reaction activity by changing the overlap of orbitals of catalysts [161].

Furthermore, one of the benefits of surface lattice strain is that the catalyst composition remains unchanged [162]. Calculations based on DFT have an advantage in forecasting the trend and influence of lattice strain on the photocatalytic performance of 2D materials in water splitting [87]. A few examples include noble metals, 2D transition-metal complexes, heterostructure, and perovskite oxides, indicating that lattice strain has a lot of potential for changing the photocatalytic performance for water splitting [89,101,163,164,165,166,167,168,169,170,171,172,173,174,175,176,177,178].

**Figure 8:**
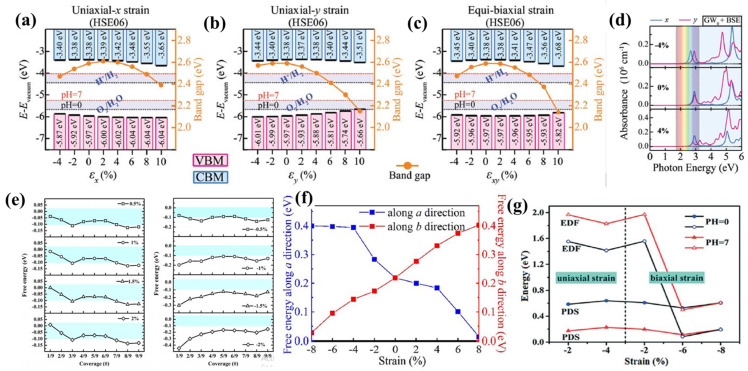
The variation of the band edge position of the VBM (depicted as pink bars) and CBM (depicted as sky blue bars) and band gap energy (depicted as orange points with orange line) computed using the HSE06 functional for h-AlN monolayer with respect to (**a**) uniaxial-x (**b**) uniaxial-y, and (**c**) equi-biaxial strains. (**d**) Variation of optical absorbance coefficient with respect to equi-biaxial strain computed using GW0 + BSE approximation. Reprinted with permission from ref. [179]. Copyright 2021 RSC. (**e**) Variation of the free energies of hydrogen adsorption with applied biaxial strain. The free energy with catalytic activity comparable to that of Pt is highlighted in light blue. Reprinted with permission from ref. [182]. Copyright 2020 ACS. (**f**) The hydrogen adsorption free energy of Cu@Sb under strain from −8% to 8%. Reprinted with permission from ref. [165]. Copyright 2020 Elsevier. (**g**) The EDF and PDS of uniaxial and biaxial strained WS2/BlueP during OER at PH = 0 and PH = 7. Reprinted with permission from ref. [166]. Copyright 2021 RSC.

The bandgap, as well as indirect-to-direct transition, can be modulated by strain engineering [179]. For example, when subjected to uniaxial and biaxial strains (compressive and tensile) in hexagonal AlN (h-AlN) monolayer, its bandgap and band edge alignment perfectly match with the water-splitting energy levels (Figure 8a–c) [179]. Besides the suitable band gap and appropriate band alignment of the h-AlN monolayer, the optical absorption capability can also be modified by strain engineering [51,52,53,54]. Figure 8d shows that the optical absorption peak of strained th-AlN has different absorption energies than the pristine th-AlN monolayer. In addition, the optical properties can also be tuned with strain engineering [152,180,181]. Importantly, Zhao et al. [182] demonstrated that applied tensile strains (0–2%) improve the HER activity of the h-B_2_O monolayer, resulting in ΔG* being close to 0 eV at low hydrogen coverage, as shown in Figure 8e. Also, the imposed compressive strain (−2 to 0%) may increase hydrogen bonding, resulting in lower ΔG* (Figure 8e).

Furthermore, the uniaxial strain (−8% to +8%) can also improve the HER activity of the α-Sb monolayer [182]. For Cu-doped α-Sb monolayer, the value of ΔG_H*_ decreases with both tensile and compressive strain (Figure 8f). This observation indicates that the HER catalytic performance of the monolayer can be promoted by applying a uniaxial strain. Besides HER, OER catalytic performance is also boosted with strain engineering for the photocatalytic process. The values of the EDF (electrochemical driving forces) at pH = 0 are more than the PDS (potential determining step) for 2% and 4% uniaxial strain. The −2%, −4% uniaxial and −2% biaxial strained WS_2_/BlueP heterostructures are thermodynamically feasible at pH = 0 for photocatalytic water-splitting (Figure 8g). In contrast, at pH = 7, all the strained heterostructures are feasible [166].

### 3.4. Intrinsic Electric Fields

An electric field can modify the photocatalyst bandgap requirement of 1.23 eV in the reaction mechanism of water splitting. Yang and colleagues first introduced a new way to breach the 1.23 eV limit and significantly boosted solar energy efficiency [84]. When an external electric field is introduced in the interface between two identical photocatalysts, all energy levels bend in the same direction, leading to the development of a nano-heterojunction with the charge distribution of the VB on one surface (001) and the CB on the other (001) surface.

In contrast, the reduction potential on the (001) surface should be below the CBM, according to this newly proposed principle. Furthermore, the internal electric field prevents excited electrons on the (001) surface from returning to the (001), encouraging the spatial separation of the photogenerated electron-hole pair [84]. Here, we discuss many designing techniques such as Janus structure, type-II heterostructure, and Z-scheme heterostructure, which can have a self-induced internal electric field, reducing the HER and OER overpotentials of 2D semiconductor to the levels that the photogenerated carriers can provide.

#### 3.4.1. Janus Structures

Researchers recently became more interested in Janus structures, a new class of 2D materials with unique properties induced by symmetry breaking and intrinsic out-of-plane polarization [183,184,185]. Because of their inherent out-of-plane mirror asymmetry, Janus monolayers have been proposed to be effective photocatalysts for water splitting and piezoelectric materials in device applications.

**Figure 9:**
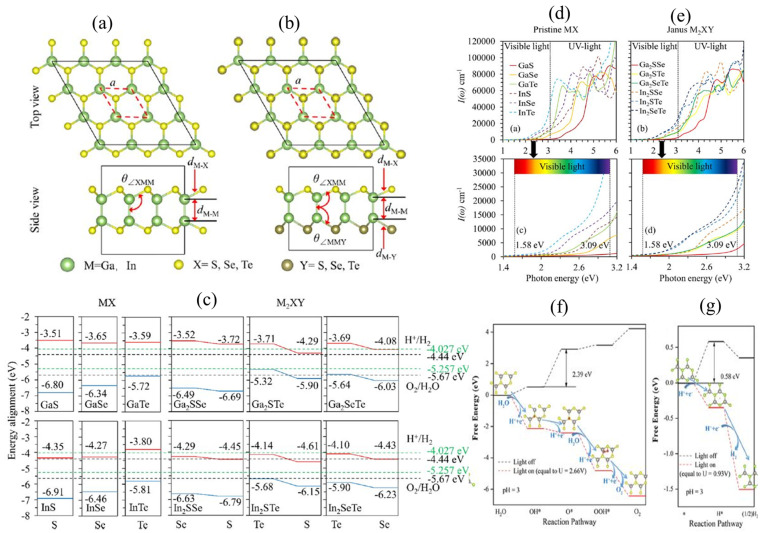
The geometric structure of the 2D (**a**) MX and (**b**) Janus M_2_XY monolayers and their (**c**) electronic band edge positions relative to the vacuum level. Reprinted with permission from ref. [186]. Copyright 2019 ACS. The optical absorption coefficients I(ω) and in the visible light region for the 2D (**d**) MX and (**e**) Janus M_2_XY monolayers. (**f**) OER and (**g**) HER at pH = 3 on the WSSe monolayer under different illumination conditions, respectively. The grey and red balls separately are the H and O atoms. Reprinted with permission from ref. [108]. Copyright 2020 ACS.

The recently synthesized asymmetric Janus TMDCs (Transition-metal dichalcogenides) inherit the advantages of two H-phase TMDCs while also providing an additional degree of flexibility to boost photocatalytic performance [184]. Figure 9a depicts the typical structure of an MX monolayer’s atomic structure, composed of two layers of vertically bonded metal atoms sandwiched between two layers of chalcogen atoms. A Janus M_2_XY monolayer is formed by substituting the bottom layer of chalcogen atoms with another chalcogen atom, as shown in Figure 9b [186]. The Janus M_2_XY and parent MX monolayers possess negative formation energies, indicating that they can be formed stably as investigated by DFT computations. Furthermore, MX and Janus M_2_XY monolayers exhibit suitable band alignments (Figure 9c), with a robust optical absorption coefficient (~3 × 10^4^/cm) in visible light and an even higher absorption coefficient (~10^5^/cm) in the near UV range (Figure 9d,e) [186].

The WSSe monolayer’s driving force for the photoexcited carriers has been investigated by Kou et al. using DFT calculation [108]. Step 2 (ΔG_O_*) has a positive free energy change, indicating that the water oxidation half-reaction on the S side cannot proceed in the dark medium. During the reaction, ΔG_O_* is the maximum, termed the OER barrier (rate-limiting potential). When the WSSe monolayer is exposed to light, photogenerated holes provide sufficient light. U_h_, which grows with increasing pH value, contrasts the situation of ΔG_O_*. Surprisingly, all of the steps in the half-reaction of water oxidation are favourable (ΔG < 0) at pH = 1–7, as shown in Figure 9f. In other words, in a light environment, the H_2_O molecules could spontaneously oxidize into O_2_ on the Janus monolayer WSSe at neutral or even acidic media (1–7 pH) [108].

On the other hand, the hydrogen reduction at pH = 0−7 cannot proceed spontaneously on the Se side without light irradiation. When U_e_ is evaluated at pH = 0, both steps are energetically favorable (ΔG < 0), indicating that the HER might occur spontaneously on the Se side in an acid media (0–5 pH) under U = 0.93 V (Figure 9g). At pH = 3, the WSSe monolayer shows optimal work conditions for catalytic reaction due to the competitive correlation between the pH requirements for limiting the catalytic reaction barriers [108].

In conclusion, the 2D Janus materials have more immense redox potentials than their parent equivalents and can derive sufficient driving force from photo-excited carriers to facilitate overall water-splitting [166,187,188,189,190,191]. Due to their adequate band gaps and excellent redox potentials, Janus Ga_2_SeTe, Ga_2_STe, Ga_2_SSe, Pd_4_S_3_Se_3_, Pd_4_S_3_Te_3_, Pd_4_Se_3_Te_3_, and Janus TM oxides monolayers such as TiSO, ZrSO, and HfSO are projected to be attractive photocatalysts [192,193,194].

#### 3.4.2. Heterojunctions

Heterojunctions have been developed as a new technique to design high-performance photocatalysts for water splitting [195,196]. There are two types of heterojunctions for photocatalytic water-splitting systems, namely type-II [197] and Z-scheme [198,199,200] heterojunctions. This proposed strategy can enhance the separation efficiency of photogenerated electrons and holes and catalytic reaction activity by occurring on different surfaces of materials.

##### Type-II van der Waals Heterostructures (vdWH)

Type-II vdWH can be constructed by layering monolayers on top of other monolayers [201,202,203]. The photogenerated electrons and holes are trapped independently in various building blocks; thus, lowering the recombination rate with more active sites. Therefore, type-II heterojunctions photocatalysis is very useful to split water photo catalytically since it avoids the unwanted surface back reactions to a large extent [40,195,196,204]. Therefore, there are three essential criteria to construct type-II heterojunctions: (i) The gap between CBM and VBM in two types of monolayers must be slightly overlapped to assure the separation of holes and electrons. (ii) Photocatalytic activity for water splitting should be present in both types of monolayers within a specific pH range. (3) The lattice parameters of two different types of monolayers must be similar enough to reduce strain and distortion in their lattices while also improving stability. A matched lattice can also be designed by combining supercells with various numbers of unit cells, for example, a 2 × 2 supercell with a 3 × 3 supercell.

**Figure 10:**
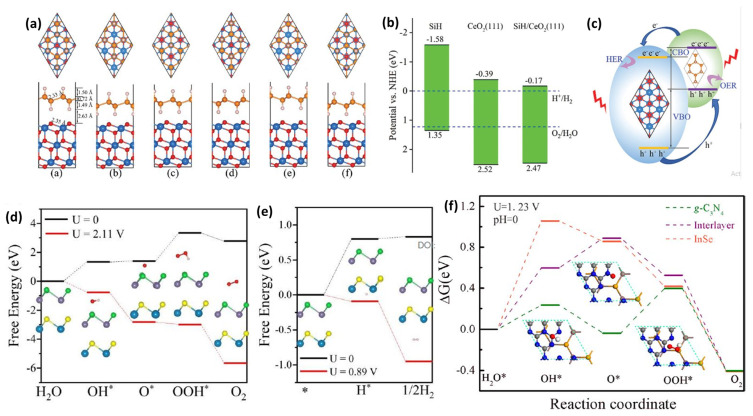
(**a**) The six different stacking patterns for 2 × 2 SiH/CeO_2_(111) heterojunctions, including top and side views. (**b**) Calculated band edge potentials versus NHE of SiH, CeO_2_(111), and SiH/CeO_2_(111) heterojunction. (**c**) Schematic diagram of charge transfer between SiH/CeO_2_(111) heterojunction layers. Reprinted with permission from ref. [216]. Copyright 2021 RSC. The free energy steps for (**d**) OER and (**e**) HER in SeGe@SSn for potential U = 2.11 V and U = 0.89 V at pH = 7. Reprinted with permission from ref. [119]. Copyright 2019 RSC. (**f**) The ΔG profiles of the OER (pH 0, U = 1.23 V) on the InSe surface, the g-C_3_N_4_ surface, and the interlayer of InSe/g-C_3_N_4_. Reprinted with permission from ref. [217]. Copyright 2019 ACS.

Various type-II heterojunctions have been reported with different materials interface stacking to show charge separation as photocatalysts for splitting water [205,206,207,208,209,210,211,212,213,214,215]. A novel SiH/CeO_2_(111) type-II heterojunction with the six stacking modes was constructed with optimized structures (Figure 10a) having a slight lattice mismatch of less than 1% [216]. The electron and hole accumulation regions formed at the heterostructure interface due to the vdW interaction between SiH and the CeO_2_(111) layer, separating the photoexcited electron-hole pairs. The band edge potentials of pristine SiH monolayer, CeO_2_(111), and SiH/CeO_2_(111) heterojunction with respect to the 4.5 eV NHE are shown in Figure 10b. All of their VBM (CBM) potentials are greater (lower) than the oxygen reduction (proton reduction) potential, suggesting that the catalytic reaction may be successfully executed under light-driven conditions [216]. 

In addition, Figure 10c shows a schematic model of charge transfer between SiH/CeO_2_(111) heterojunction layers, which clarifies the mechanism of photocatalytic water-splitting. Firstly, light excites VB electrons to CB of the SiH and CeO_2_(111) layers, while holes stay in VB. Photogenerated electrons and holes tend to gather on the CeO_2_(111) and SiH monolayers, respectively, thereby delaying photogenerated carrier recombination. As a result, OER and HER reactions may be carried out efficiently on various monolayers [216].

Without any light irradiation (U = 0), the SeGe@SSn heterostructure carried out the OER reaction, which resulted in a rate-determining step of 1.94 eV (Figure 10d). While the photogenerated holes of SeGe@SSn offer the additional potential (U = 2.11 V) for the irradiated sample in the neutral solution [119]. All steps are energetically favorable (ΔG < 0), implying that under light irradiation at pH = 7, all oxidation reactions can proceed spontaneously without any barriers, which is highly appealing for practical applications. On the other hand, only two hydrogen reduction processes are required, as shown in Figure 10e. The fact that the extrinsic potential provided by photogenerated electrons (U = 0.89 V) can be generated under light irradiation makes both of these steps exothermic. Thus, it is reasonable to assume SeGe@SSn to be capable of photocatalytic water-splitting without using a sacrificial agent when exposed to light [119].

In addition, the computed ΔG for the OER process at U = 1.23 V for the InSe/g-C_3_N_4_ heterostructure are illustrated in Figure 10f. The third reaction step has the maximum ΔG of 0.433 eV on the g-C_3_N_4_ side, while the highest ΔG value of 1.057 eV on the InSe side and 0.596 eV at the InSe/g-C_3_N_4_ interface, respectively, for the dissociation of H_2_O to OH*. The increasing trend of ΔG value for the H_2_O to OH* step reveals that the InSe/g-C_3_N_4_ heterojunction catalyst has a high active water adsorption energy on all three sides, preventing water dissociation OH* intermediates species, resulting in slow OER kinetics [217]. Due to the separation of photogenerated electron-hole pairs, reaction kinetics, and optical absorption, the heterostructure is widely thought to be a viable solution to large overpotential for HER and OER. As a result, numerous 2D Janus vdW heterojunctions have been designed, such as MoSTe/C_3_N_4_ [218], ZnO/WSSe [219], MoSSe/SiC [220], GeC/MoSSe [221], GaS0.5Se0.5/Arsenene [222], MoSSe/AlN [223], MoSSe/GaN [223], GeC/WSSe [221], WSSe–SiC [220], MoSSe/WSSe [224], MoSSe/C_3_N_4_ [218], and ZnO/MoSSe [219] heterostructures for photocatalytic water-splitting.

##### Z-Scheme Heterostructures

**Figure 11:**
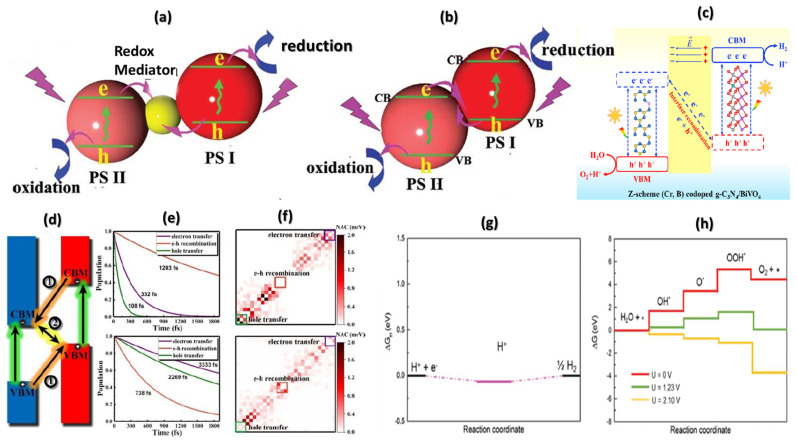
Schematic illustrations of (**a**) ideal Z-scheme system and (**b**) direct Z-scheme system. Reprinted with permission from ref. [226]. Copyright 2015 Wiley-VCH. (**c**) Schematic illustrations of the reaction mechanism for photocatalytic water-splitting and photocatalytic mechanism. Reprinted with permission from ref. [227]. Copyright 2020 Elsevier. (**d**) Schematic diagram of the photo-excitation and charge transfer dynamics at the MoSSe/WSeTe interface (**e**) The electron transfer, hole transfer, and e-h recombination dynamics of MoSSe/WSeTe heterostructure with Te-Se and Te-S stacking. (**f**) Averaged values of NAC between different states for Te-Se and Te-S stacking. Reprinted with permission from ref. [240]. Copyright 2019 RSC. (**g**) ΔG_H_ as a function of reaction coordinate on Are layer in the PtS_2_/Are vdW heterostructure. (**h**) The free energy diagram for OER on PtS_2_ layer in the heterostructure at U = 0 V, 1.23 V, 2.10 V potential. Reprinted with permission from ref. [110]. Copyright 2020 RSC.

In contrast to one-step excitation, the Z-scheme photocatalytic mechanism [198,200] is a two-step excitation system motivated by natural photosynthesis in green plants [225]. The ideal Z-scheme photocatalysts comprise three components as two different photosystems are connected by the acceptor/donor pair (shuttle redox mediator), as schematically illustrated in Figure 11a. In contrast, the separate components of these Z-scheme photocatalysts systems for the catalytic reaction are inappropriate for overall water decomposition. On the other hand, redox mediators in Z-scheme photocatalysts may cause unwanted reverse reactions and prevent optical absorption [226]. Therefore, as schematically illustrated in Figure 11b, mediator-free systems are direct Z-scheme systems. Hence, direct Z-scheme photocatalysts for overall water breakdown have sparked much interest because of their remarkable redox capability and effective separation of photogenerated electron-hole pairs [227,228,229,230,231,232,233,234,235,236,237,238].

The charge redistribution and generation of the built-in electric field across the heterostructure interface are driven by the difference in work function (W) between the adjacent monolayers [239].

Figure 11c depicts the photocatalytic mechanism of pure, Cr, B doped, and co-doped g-C_3_N_4_/BiVO_4_ interfaces [227]. The face-to-face g-C_3_N_4_/BiVO_4_ 2D materials of g-C_3_N_4_ and BiVO_4_ (010) surface can initiate an electric field, encouraging interface charge transfer and preventing electron-hole pair recombination resulting in a negative charge on the g-C_3_N_4_ monolayer and a positive charge on the BiVO_4_ (010) surface. Figure 11d is a schematic diagram of the photo-excitation and charge transfer mechanism at the MoSSe/WSeTe interface [240]. In theory, if process 1 has a longer lifetime than process 2, photoexcited electrons at MoSSe’s CBM tend to recombine with holes at WSeTe’s VBM, generating a Z-scheme path. While process 2 has a longer lifetime than process 1, the photoexcited electrons and holes split fast and remain at MoSSe and WSeTe, respectively, operating as a conventional type II channel.

The electron transfer, hole transfer, and e-h recombination dynamics of MoSSe/WSeTe hetero-structure with Te-Se and Te-S stacking are shown in Figure 11e [241,242]. The rapid charge separation demonstrated that the charge transfer in Te-Se stacking follows the typical type-II path. At the same time, the e-h recombination makes the charge transfer facilitation in the Z-scheme path for the Te-S stacking [240]. In addition, the type II path for Te-Se stacking and the Z-scheme pathway for Te-S stacking is also confirmed by the averaged nonadiabatic coupling (NAC) (Figure 11f). The Z-scheme mechanism benefits from the enormous long carrier lifetime and photoinduced charge density to increase the reduction and oxidation reaction rates. For example, the PtS_2_/Arsenene vdW heterostructure can split the water into hydrogen and oxygen by direct Z-scheme [110]. Figure 11g shows the ΔG_H_ is as low as –0.487 eV, with the most stable adsorption site for HER on the arsenene layer. As a result, the PtS_2_/Arsenene vdW heterostructure is an intriguing HER catalyst candidate. In OER, under U = 0, the rate-limiting step (2 eV) is the production of OH* by H_2_O adsorption on the PtS_2_ layer. The ΔG steps for OER change for this reaction step are decreased at an external potential of 1.23 V, as represented by cyan lines in Figure 11h. Moreover, all reaction steps are energetically favorable (ΔG < 0) in free energy under 2.10 V potential field, as shown by yellow lines (Figure 11h), indicating that OER has a lower exothermic reaction than other 2D photocatalysts [97].

### 3.5. Multilayer Configurations

The thickness heavily influences the electronic structures of 2D layered semiconductors [243,244,245,246,247]. The thickness-dependent electronic characteristics severely limit the potential applications of 2D stacked semiconductors as photocatalysts. For example, the bandgap with conduction and valence band locations shift dramatically from a single layer to a few layers, as seen in MoS_2_ and phosphorene layers [248,249,250]. As a result, it is critical to find 2D photocatalysts with appropriate thickness-independent band gaps and band edge alignment with high mobility [36,251]. However, a few single photocatalysts can spontaneously fulfill high solar energy conversion efficiency and photocatalytic redox processes.

**Figure 12:**
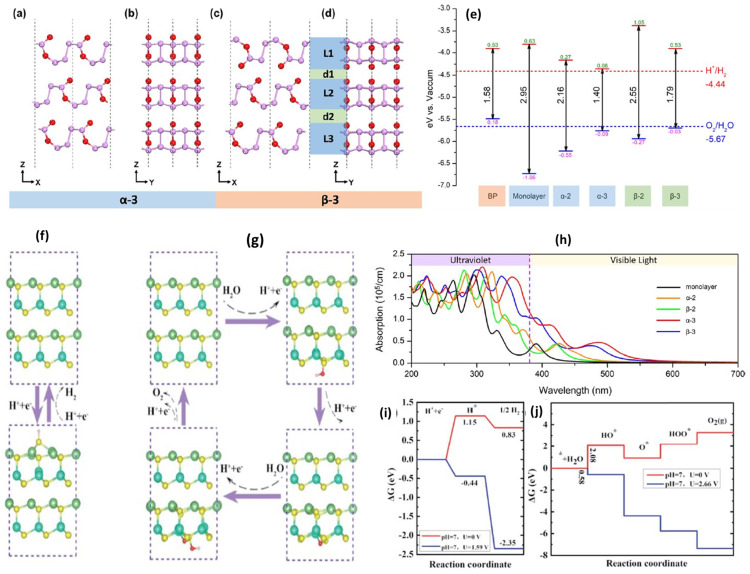
Geometric structure of few-layer P_4_O_2_ systems. The side views of (**a**,**b**) α-three-layer P_4_O_2_ in α packing manner) and (**c**,**d**) β-3 (three-layer P_4_O_2_ in β packing manner). (**e**) Band edge positions for few-layer P_4_O_2_ and monolayer BP. Reprinted with permission from ref. [252]. Copyright 2019 ACS. The proposed photocatalytic pathways of intermediate species for the (**f**) HER and (**g**) OER in the LiGaS_2_ bilayer. (**h**) Optical absorption spectrum of few-layer P4O_2_ calculated by the GW + BSE approach. Free energy profile for the (**i**) HER and (**j**) OER pathway in the LiGaS_2_ bilayer at different voltages. Reprinted with permission from ref. [97]. Copyright 2019 RSC.

Li et al. [252] predict the optoelectronic properties of few-layer P_4_O_2_ systems using DFT computation. A three-layer P_4_O_2_ with standard (α-3) packing is an attractive option for photocatalytic water-splitting because it facilitates the required optoelectronic properties (Figure 12a–d). The bandgap and band edge positions of monolayers α-2, α-3 and β-2, β-3, surpass the redox potential of water splitting. As a result, they could be good candidates for photocatalysts that do not require an external potential (Figure 12e). More intriguingly, the CBM of α-3 is 0.08 eV larger than the reduction level of H^+^/H_2_O, whereas the VBM of α-3 is 0.09 eV lower than the oxidation level of O_2_/H_2_O. As a result, α-3 might be considered the most efficient photocatalysts for overall water-splitting processes [252]. Furthermore, all five systems have significant optical absorption in visible and UV spectral regions (Figure 12h). The absorption coefficients of α-3 and β-3 are substantially more effective than those of the other three systems due to interlayer interaction [252].

Furthermore, Qi et al. [97] proposed experimentally feasible 2D LiMX2 (M = Al, Ga, In; X = S, Se, Te) bilayers to be ideal for photocatalytic water-splitting using first-principles calculations. The hydrogen atom prefers to bind to the S site, as seen in Figure 12f. However, after hydrogen desorption, such a deformed surface will be restored to its original structure. As shown in Figure 12i, free energy change calculations demonstrate that H adsorption on the LiGaS_2_ bilayer at U_e_ = 0 is uphill with ΔG =1.15 eV. So, it cannot proceed spontaneously.

Interestingly, all HER steps become exothermic reactions when exposed to the potential (U_e_ = 1.59 V) produced by photogenerated electrons (Figure 12i). On the other hand, Figure 12g depicts the photocatalytic mechanism for OER corresponding with their free energy at U_h_ = 0 (Figure 12j). The first step is the rate-limiting step, with a ΔG barrier of 2.08 eV. The photogenerated hole U_h_ = 2.66 V potential can surpass this step. Therefore, the ΔG plot shows a downward trend at this voltage, indicating that the OER can drive under these conditions. The findings show that the photogenerated carriers in the LiGaS_2_ bilayer have sufficient photocatalytic redox potential to drive the catalytic reaction in pure water [97].

## 4. Edge-Modification in Nanoribbons

**Figure 13:**
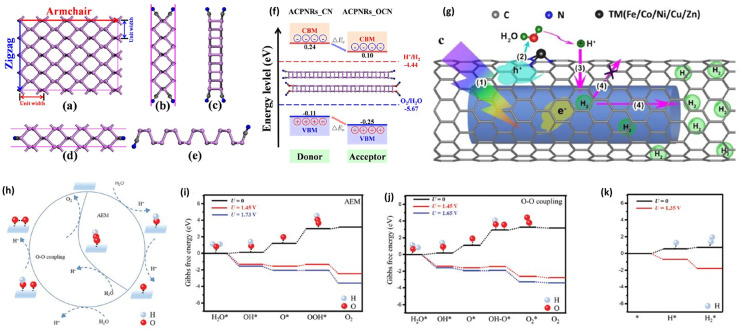
Geometric structures of pristine phosphorene and edge modified PNRs: (**a**) phosphorene in a 4 × 4 supercell; (**b**,**c**) top and side views of ACPNRs passivated by nitrile (CN) functional group in ribbon width of 4 (ACPNR4_CN); (**d**,**e**) top and side views of ZZPNRs by nitrile (CN) active group in ribbon width of 4 (ZZPNR4_CN). (**f**) Schematic illustration of type-II donor−acceptor band alignments edge modified PNR heterobilayers. Reprinted with permission from ref. [265]. Copyright 2017 ACS. (**g**) Photocatalytic hydrogen generation and collection scheme. Reprinted with permission from ref. [268]. Copyright 2019 ACS. (**h**) Reaction mechanism for two possible mechanisms of the OER via single-site or dual-site reactions on F-10BN_ZZ-GDYNR1 (GDYNR stands for graphdiyne nanoribbons). Reaction energy pathways of (**i**) the OER following the AEM mechanism, (**j**) the OER through the O–O coupling mechanism, and (**k**) the HER on F-10BN_ZZ-GDYNR1 under different electrode potentials U relative to SHE at pH = 7. Reprinted with permission from ref. [269]. Copyright 2020 RSC.

Due to the quantum confinement, nanostructures that lower the dimensionality of 2D materials to nanoribbons and quantum dots have tunable electronic properties [253,254,255,256,257,258]. Designing nanoribbons (NRs) for photocatalytic water-splitting by modifying their edges is a new technique for enhancing photocatalytic efficiency [259,260]. According to a quantitative analysis of the Gibbs free energy of hydrogen (H*) adsorption/desorption, edges play a dominating role in triggering the photocatalytic process [261,262,263]. Yang et al. [264] present a novel approach for edge-modified phosphorene nanoribbons (PNRs) as very effective photocatalysts. They find that pseudo-halogen passivated PNRs have the desired band edge positions urged by the edge electric dipole layer, preserving the intrinsic optoelectronic properties of phosphorene [265,266]. Figure 13a–e shows the armchair (AC) and zigzag (ZZ) PNRs passivated by the CN functional group. It is worth noting that edge passivation can result in polar covalent bonds forming along the edges of PNRs [264]. A potential jump shifts to all single-particle energy levels and the band edge energy levels. As a result, the phosphorene’s band edge energy levels can be shifted downward employing polar covalent bonds in the form of P^+^ − X^−^ where X represents the atom or a chemical group. This vital edge decoration affects the CBM and VBM levels of edge-modified PNRs, allowing for the design of efficient photocatalysts [265]. In addition, the PNR heterobilayer is designed, which consists of two different types of edge-modified PNRs, as shown in Figure 13f. Heterobilayer acts as a type-II donor-acceptor interface because of the built-in potential induced by the edge dipole layers. This heterojunction type can promote carrier transport and separation at the heterobilayer interface. VBM and CBM states are anticipated to be localized inside discrete donor and acceptor regions due to such an induced potential. Localization of the VBM and CBM states improves charge separation and allows carrier collection easier [267].

Moreover, Yang et al. [268] designed a photocatalytic hydrogen generating system using carbon nanotube (CNT) and carbon-nitride double-walled nanotube systems (CNNT), in which CNNT is caged in CNT (Figure 13g). To improve water dissociation, they used mono dispersed TMN3 (TM = Fe/Co/Ni/Cu/Zn) sites embedded in CNT, abbreviated as CNTFCNNT. Figure 13g depicts the photocatalytic hydrogen generation and collection scheme: (i) electrons in CNNT and holes in CNT, (ii) water decomposes into a proton and oxygen-containing groups due to energetic holes, (iii) when protons arrive at CNNT, they are converted to hydrogen atoms and (iv) H_2_ production can be entirely isolated from oxygen-containing species, thanks to CNT’s selective permeability of protons. Finally, H_2_ molecules should diffuse in the gap between two nanotubes, allowing safe delivery [268].

On the other hand, doping of graphdiyne nanoribbons (GDYNRs) with BN pairs can result in an appropriate band structure for accelerating catalytic reactions [270,271]. The F-10BN ZZ-GDYNR1 and F-8BN ACGDYNR1 explore the thermodynamics of the OER half-reaction, followed by the adsorbate evolution mechanism (AEM), O–O coupling, and the HER mechanisms (Figure 13h). 

Alternatively, the OER can continue via the O–O coupling pathway and other OH* formed by the second dissociation of H_2_O, which couples with a co-adsorbed O* at a nearby site (Figure 13i). It led to an OO* species formation and releasing an (H+–e) pair with a ΔG of 1.65 eV. The desorption of OO* creates an O_2_ gaseous molecule in the last stage because the rate-determining phase has a greater negative ΔG. The O–O the coupling mechanism is significantly more likely to occur than the AEM mechanism (Figure 13j) [269]. Therefore, the F-10BN ZZ-GDYNR1 photocatalyst possesses an unfavourable ΔG at pH = 7. At U = 0, ΔG for the production of H_2_ molecules from the H* species is 0.19 eV for the HER, as illustrated in Figure 13k. After shifting the energy by an external potential given by photogenerated electrons (i.e., Ue = 1.35 V), the HER’s elementary steps become gradually smaller than zero. The results presented by Xu et al. demonstrate that F-10BN_ZZ-GDYNR1 is a viable candidate for use as a metal-free photocatalyst in overall water [269].

## 5. The Effect of pH in Overall Water Splitting

It has been shown that altering the redox potential of water splitting can improve the photocatalytic activity of semiconductors (generally by changing the pH) [272,273]. The catalytic reaction mechanism is highly dependent on environmental conditions [274]. In acidic and alkaline environments, the half-reaction on the surface for water oxidation is different. The mechanism of OER under acidic environments has been extensively studied using first-principles studies [89,118,275,276]. The ΔG of H^+^ + e^−^ at a pH other than 0 can be formally addressed using the computational hydrogen electrode (CHE) approach established by Bagger et al. [277] by using the reversible hydrogen electrode (RHE) as the reference electrode and standard hydrogen electrode (SHE) for pH = 0. The two reference potentials $U_{\mathrm{RHE}}$ and $U_{\mathrm{SHE}}$ can be linked as ${\mathrm{eU}}_{\mathrm{RHE}}={\mathrm{eU}}_{\mathrm{SHE}}-k_{B}{\mathrm{Tlna}}_{H^{+}}={\mathrm{eU}}_{\mathrm{SHE}}+k_{B}T\;\times \mathrm{pH}\times \ln10$. The $a_{H^{+}}$ is the activity of the H^+^ ions in solution at T = 298.15 K [278,279]. Notably, fewer DFT studies for water oxidation in the alkaline medium were reported in the literature [279,280,281]. The reduction and oxidation potential for water splitting under the influence of pH of the medium is given as [105]

(12) $$E_{H^{+}/H_{2}}=-4.44\;\mathrm{eV}+\mathrm{pH}-0.059\;\mathrm{eV}$$

(13) $$E_{O_{2}/H_{2}O}=-5.67\;\mathrm{eV}+\mathrm{pH}-0.059\;\mathrm{eV}$$

**Figure 14:**
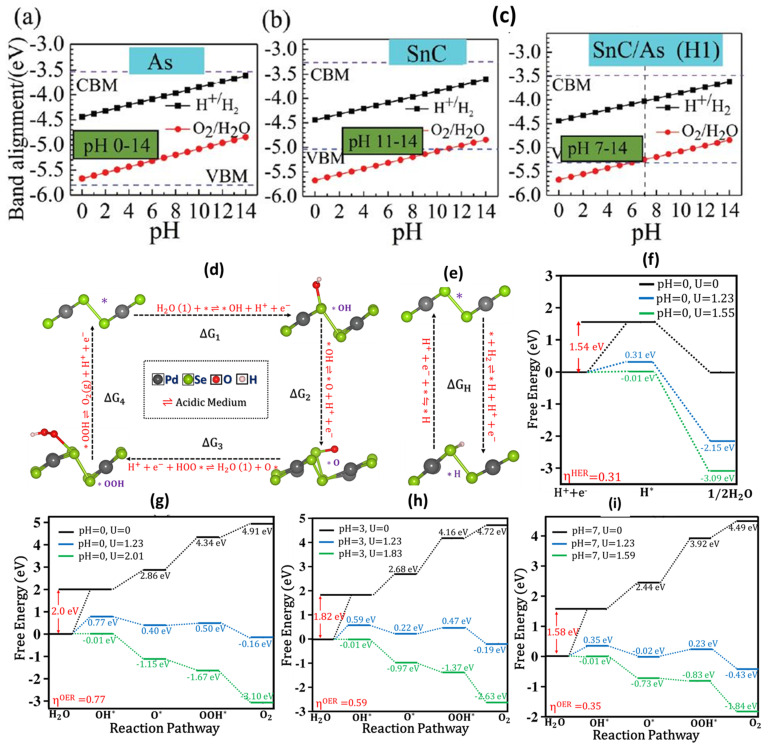
The band edge alignment of (**a**) As, (**b**) SnC, and (**c**) SnC/As heterostructure with the redox potentials of water splitting as a function of pH. Reprinted with permission from ref. [282]. Copyright 2020 RSC. Proposed photocatalytic pathways and the atomic configuration of absorbed intermediates species during the (**d**) OER and (**e**) HER on the β-PdSe_2_ monolayer in an acidic medium. The free-energy changes of hydrogen reduction at (**f**) pH = 0 and oxygen evolution at (**g**) pH = 0 (**h**) pH = 3 (**i**) pH = 7 on the β-PdSe_2_ monolayer. Reprinted with permission from ref. [105]. Copyright 2021 RSC.

Li et al. [282] have investigated the effect of pH on the photocatalytic activity of SnC and As monolayers, as well as the SnC/As heterojunction [282]. The reduction and oxidation potentials are between the band edges of the As monolayer, with a pH (0 to 14) function, as illustrated in Figure 14a. As a result, the As-monolayer meets the fundamental criteria of photocatalytic total water splitting, but SnC fails to meet this condition (Figure 14b). Furthermore, the alkaline conditions are appropriate for photocatalytic water-splitting in H1 configuration of SnC/As heterojunction (Figure 14c).

### 5.1. Catalysis in Acidic Media

Our group has recently demonstrated that the overall water-splitting can occur simultaneously and spontaneously on β-PdSe_2_ monolayer in all media conditions [105]. Figure 14d depicts the four-electron (4e) reaction pathway, followed by adsorbed intermediates forming on a β-PdSe_2_ monolayer. At U = 0 V, the first step of the β-PdSe_2_ monolayer combines with a proton and electron to produce a H* intermediate with uphill ΔG of 1.54 eV (Figure 14e). In contrast, with U = 1.23 V, the first step is uphill, and the second step is downhill. While at U = 1.55 V, the free energy diagram demonstrated that all reaction steps were downhill. As a result, the calculated HER overpotential (η^HER^) for a β-PdSe_2_ monolayer at pH = 0 is 0.31 V (Figure 14f) [105].

In an acidic medium, the ΔG of elementary steps in the dark (U = 0) and light (U = 1.23 eV, 2.01 eV) radiated conditions show that two reaction steps are uphill and the other two steps are downhill at the equilibrium potential (1.23 V) for pH = 0 to 7. While at U = 2.01 V, the free energy profile revealed all reaction steps exergonic for pH = 0 to 7. As a result, the computed OER overpotential (η^OER^) for a β-PdSe_2_ monolayer at pH = 0 was 0.77 V, as shown in Figure 14g. 

The free energy profile for OER at pH = 3 (Figure 14h) reveals that the overpotential (η^OER^) is lowered to 0.59 V (pH = 3) from 0.77 V (pH = 0). In addition, the free energy profile for the β-PdSe_2_ monolayer in a neutral environment showed that all reaction steps were downhill at U = 1.59 V for pH = 7 (Figure 14i).

### 5.2. Catalysis in Alkaline Media

**Figure 15:**
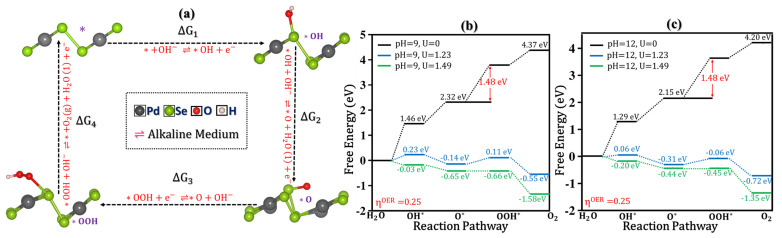
Proposed photocatalytic pathways and the atomic configuration of absorbed intermediates species during the (**a**) OER on the β-PdSe_2_ monolayer in an alkaline medium. Free energy diagram for OER at (**b**) pH = 9 (**c**) pH = 12 on β-PdSe_2_ monolayer. Reprinted with permission from ref. [105]. Copyright 2021 RSC.

Figure 15a shows proposed photocatalytic pathways and the atomic configuration of absorbed intermediates species during the OER on the β-PdSe_2_ monolayer in an alkaline medium [105]. When U = 1.49 V, the ΔG of the oxidation reaction for β-PdSe_2_ monolayer in an alkaline environment (pH = 9, 12) becomes energetically favorable with ΔG < 0. However, at U = 0, the species OOH* formation (+1.48 eV) is the rate-limiting step, which is further decreased to 0.25 eV at an equilibrium potential of 1.23 V at pH = 9, 12 (Figure 15b,c). Furthermore, the overpotential (OER) of the β-PdSe_2_ monolayer is 0.25 V, which is substantially lower than extensively investigated photocatalysts β-SnSe (0.28 V) [89] and Ni/graphene (0.35 V) [275] g-C_3_N_4_ (0.43 V) [283], β-GeSe (0.49 V) [89], C_2_N/GaTe (1.47 V), and C_2_N/InTe (0.94 V) [116]. These theoretical results demonstrate that the experimentally achievable β-PdSe_2_ 2D material is highly efficient for photocatalytic water-splitting in any environmental medium.

## 6. Conclusions, Perspectives, and Challenges

The emergence of environmental and energy challenges has seriously hindered global development and progress. Photocatalytic water-splitting to produce hydrogen opens up a new way to develop new energy sources. In the last few decades, extensive efforts have been devoted to constructing various photocatalytic 2D semiconductors due to their exceptional optical features and potential uses in the energy and sensing domains. Since first-principles methods can predict the intrinsic photocatalyst properties and be employed to screen the photocatalytic systems reliably and efficiently, this review focuses on the theoretical perspective of photocatalysis with 2D materials, especially the thermodynamics and kinetics aspect involved in the water-splitting mechanism. 

We discussed the computational modelling and the reaction kinetic for photocatalytic water-splitting of very recently emerged next-generation 2D materials to show the fundamental principles of reactions occurring on the surface of a photocatalyst. Notably, particular emphasis has been placed on various strategies implemented in specific examples to address issues associated with redox reactions (HER and OER) on the surface of 2D semiconductor photocatalysts. Various effective methods have been adopted to optimize the catalytic performance at the electronic and free energy levels. The defect and strain engineering can significantly improve catalysts’ HER/OER activity to the low reaction energy barrier. The formation of a self-induced internal electric field in Janus structures and heterostructures is another efficient method to improve reaction catalytic performance via accelerated electron transfer. In addition, the photocatalytic performance of 2D materials can also be enhanced by operational conditions such as surface modification and the pH by overcoming large overpotential to trigger the overall water-splitting reactions.

However, challenges still exist, and several crucial bottlenecks must be addressed, including (i) thermodynamics of photocatalytic technique such as energetic requirements and band edge alignment relevant to the HER and OER potential must be thoroughly understood. The migratory behaviors of electrons and holes from substrates receive little attention, even though they considerably impact photocatalytic performance; (ii) photoexcitation of a 2D semiconductor photocatalyst results in photogenerated electrons and holes, inducing the photocatalyst to be excited. The feature of the excited state and the mechanism for the energy barrier lowering by light irradiation remain unknown. As a result, understanding the dynamics of excited electrons is crucial to comprehending the overall photocatalytic process; (iii) many parameters, such as trapping, inelastic and elastic electron-phonon coupling, charge localization, and thermal disorder, influence charge and energy transfer and relaxation processes need to be thoroughly addressed; (iv) additional research in catalyst dependence on pH, edge modification, and multilayer configurations is required to tune the reaction catalyst to improve visible light absorption with minimum charge separation; (v) previously reported theoretical data have been based on the traditional DFT approach, whereas the illumination of photocatalytic kinetic research is still understudied due to its technological complexity. Understanding long-term stability and access to excited state dynamic simulations that could provide kinetic parameters are critical developments needed to comprehend photocatalyst performance.

Because of rapid achievements and a broad understanding of 2D materials in various collaborative research and development sectors in the last decade, it is now an absolute delight to do photocatalysis research on these materials. At the same time, it is possible that utilizing absorbed solar energy might significantly reduce the demand for fossil fuels for human progress while also lowering environmental pollution. Consequently, photocatalysts are becoming increasingly significant, and we must increase the performance of existing photocatalysts and develop new engineering methodologies to exploit innovative photocatalysts in a better way. This review may be helpful to reduce the gap between theory and experiments to understand the photocatalytic performance of 2D materials.

## Data Availability

Not applicable.
